# The Case of Dr. Baker—Murder—Monomania

**Published:** 1845-10

**Authors:** 


### THE CASE OF DR. BAKER—MURDER—MONOMANIA.

Dr. Abner Baker, a reputable physician, of the most respectable family and connexions, killed his brother-in-law, Daniel Bates, a highly esteemed citizen of Clay county, in this State, about a year ago, and after an examination before the usual tribunal in such cases was discharged on the grounds of lunacy. He spent the winter in Havana, and while absent the Grand Jury found a verdict against him of murder. On his return in the spring his friends gave him up; he was tried at a special term of the Circuit Court, of Clay county, in June last, and found guilty. The plea of *monomania* was set up in his defence, and the testimony to that effect of several physicians, among whom was the late Professor Richardson, was clear and decided. The Governor of the Commonwealth has been petitioned to pardon on that ground, but has refused to grant the prayer. We are assured that nine out of twelve of the jury are among the petitioners, and that they express a full conviction that Baker is a monomaniac. As far as we are informed, every physician who has heard or read the testimony in the case is of the same opinion. At the request of a brother of Dr. Baker we examined, some weeks ago, a transcript of the evidence, and from what was submitted to us we did not entertain a single doubt as to his insanity. The same record was read by several of our medical friends who concurred fully with us in this opinion, and the result was that we expressed to the Governor our belief that the unfortunate man was a fitter subject for the mad-house than the gibbet. We do not know how nearly this sentiment is universal among the medical men who have investigated the case, nor are we going to say how far the opinions of such witnesses ought to prevail with the Executive; but we suppose there is no arrogance in saying, that, as to a question of insanity, no men in the community are as competent judges as physicians. We do hope that the Governor will, at least, still further extend the respite which he has granted, and that all doubt of the lunacy of the prisoner will be removed from the public mind, if indeed he is not insane, before he is made to pay this dread penalty. The moral effect of his execution while such a doubt hangs over the case, we are thoroughly persuaded, would be most injurious.

